# Ink‐Extrusion 3D Printing and Silicide Coating of HfNbTaTiZr Refractory High‐Entropy Alloy for Extreme Temperature Applications

**DOI:** 10.1002/advs.202309693

**Published:** 2024-02-28

**Authors:** Dingchang Zhang, Ya‐Chu Hsu, David C. Dunand

**Affiliations:** ^1^ Department of Materials Science and Engineering McCormick School of Engineering Northwestern University 2220 Campus Drive Evanston IL 60208 USA

**Keywords:** additive manufacturing, direct ink writing, oxidation, refractory high‐entropy alloy, silicide coating

## Abstract

An oxygen‐resistant refractory high‐entropy alloy is synthesized in microlattice or bulk form by 3D ink‐extrusion printing, interdiffusion, and silicide coating. Additive manufacturing of equiatomic HfNbTaTiZr is implemented by extruding inks containing hydride powders, de‐binding under H_2_, and sintering under vacuum. The sequential decomposition of hydride powders (HfH_2_+NbH+TaH_0.5_+TiH_2_+ZrH_2_) is followed by in situ X‐ray diffraction. Upon sintering at 1400 °C for 18 h, a nearly fully densified, equiatomic HfNbTaTiZr alloy is synthesized; on slow cooling, both α‐HCP and β‐BCC phases are formed, but on quenching, a metastable single β‐BCC phase is obtained. Printed and sintered HfNbTaTiZr alloys with ≈1 wt.% O shows excellent mechanical properties at high temperatures. Oxidation resistance is achieved by silicide coating via pack cementation. A small‐size lattice‐core sandwich is fabricated and tested with high‐temperature flames to demonstrate the versatility of this sequential approach (printing, sintering, and siliconizing) for high‐temperature, high‐stress applications of refractory high‐entropy alloys.

## Graphical abstract

This work demonstrates that an oxygen‐sensitive refractory high‐entropy alloy (RHEA) can achieve architecture freedom and good oxidation resistance by a series of simple processing steps: i) 3D ink‐extrusion printing, ii) interdiffusion‐sintering, and iii) silicide coating. This robust route unlocks rapid prototyping of RHEA parts and is applicable to a large variety of possible alloy compositions.

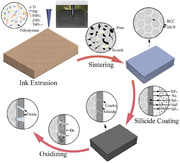

## 1 Introduction

Refractory high‐entropy alloys (RHEA, also called multi‐principal‐element refractory alloys) comprise five or more principal elements from the refractory transition metals (group 4–6 elements: Zr, Hf, V, Nb, Ta, Mo, and W, often with Ti and Cr also considered),^[^ ^1^, ^2^, ^3^ ^]^ with structural applications under extreme temperature conditions, beyond those where nickel‐based superalloys can be used.^[^ ^4^ ^]^ Additive manufacturing (AM) of RHEAs can facilitate the exploration of possible applications for RHEA via rapid prototyping of parts with complex shapes with high powder feedstock utilization. Recently, laser melting deposition (LMD) was used to fabricate NbTaTiZr,^[^ ^5^ ^]^ HfNbTiZr,^[^ ^6^ ^]^ HfNbTaTiZr,^[^ ^7^, ^8^ ^]^ and MoNbTaW^[^ ^9^, ^10^, ^11^ ^]^ quaternary and quinary refractory concentrated alloys. However, the feature resolution of LMD is relatively low, as the size of the melt pool is as large as ≈3 mm.^[^ ^7^, ^8^ ^]^ In addition, no complex parts have been demonstrated for these refractory alloys by using LMD. Laser powder bed fusion (LPBF) is another additive manufacturing method that was used to fabricate MoNbTaVW^[^ ^12^ ^]^ and MoNbTaX (X = Ti, Ni, Ti_0.5_Ni_0.5_)^[^ ^13^ ^]^ refractory alloys. However, cracking usually occurs from significant thermal stresses caused by the very fast cooling rate during the solidification of the melt pool.^[^ ^12^, ^13^ ^]^

An alternate AM method is 3D ink‐extrusion printing (material extrusion), which can extrude powder‐loaded inks, layer by layer, at room temperature to fabricate complex green parts with micro‐features, which are then densified by sintering.^[^ ^14^ ^]^ Isothermal sintering allows for very low thermal stresses preventing the associated cracking observed in LPBF. Also, sintered microstructures have no crystallographic texture, unlike LMD or LPBF alloys. Another advantage is that the printability of the ink depends solely on its rheology and does not necessitate the spherical shape and large powder sizes required for powder‐bed‐based AM methods,^[^ ^15^, ^16^ ^]^ including binder jetting.^[^ ^17^ ^]^ Furthermore, atomization of alloyed powders can be avoided by using elemental powder blends, and rapid sintering can be achieved by using fine powders with irregular shapes. Previously, 3D ink‐extrusion printing has successfully fabricated strong and ductile equiatomic lower‐melting alloys such as CoCrFeNi,^[^ ^18^ ^]^ CoCrCuFeNi,^[^ ^19^ ^]^ and CoCrFeMnNi^[^ ^20^ ^]^ HEA microlattices, using elemental or prealloyed powders. We are not aware of any published work on 3D ink‐extrusion printing of RHEA, except for Zhao et al. who used 3D ink‐extrusion printing to fabricate equiatomic NbTaTiZr scaffolds, which showed good biocompatibility and met the requirements for orthopedic implants.^[^ ^21^ ^]^

In the cast state, equiatomic HfNbTaTiZr shows good tensile ductility at room temperature (8–16%^[^ ^22^, ^23^ ^]^) and excellent strength at high temperatures^[^ ^24^ ^]^ (e.g., yield strength of 163–530 MPa at 1000 °C for strain rates of 10^−4^–10^−2^ s^−1^).^[^ ^25^ ^]^ However, the poor oxygen resistance of HfNbTaTiZr prevents most practical applications at high temperatures.^[^ ^26^, ^27^ ^]^ Coating with stable oxide formers (e.g., Al, Si, and Cr) is often used to improve the oxidation resistance of alloys: for example, Saad et al. coated Hf_0.5_Nb_0.5_Ta_0.5_Ti_1.5_Zr with an aluminide layer, which prevented the pesting behavior (disintegration into powders) when exposed to air at 800 °C for 5 h.^[^ ^28^ ^]^ To our knowledge, silicide coatings have not been studied for equiatomic HfNbTaTiZr, but they have been used to coat MoNbTaW,^[^ ^29^ ^]^ MoNbTaVW,^[^ ^30^ ^]^ and AlMo_0.5_NbTa_0.5_TiZr^[^ ^31^ ^]^; they have the advantage of forming a dense SiO_2_‐rich scale during oxidation, which can flow and heal cracks at elevated temperatures.^[^ ^30^ ^]^

In this research, we demonstrate that HfNbTaTiZr can be 3D ink‐extrusion printed from inks containing a blend of metallic and hydride powders. In situ X‐ray diffraction is used to study the sequential decomposition and metallic phase evolution of the extruded powder blend when heated under a vacuum. Microstructural evolution is studied for a range of sintering times at 1400 °C. The mechanical properties of these sintered HfNbTaTiZr bulk and 3D‐printed lattice samples are measured in compression at high temperatures. A silicide coating is deposited via cementation, and the improvement in oxidation resistance is examined. Finally, a miniaturized demonstration item with a complex shape – a lattice‐core sandwich – is printed, sintered, and tested to demonstrate the rapid prototyping capability of 3D ink‐extrusion printing for RHEA.

## 2 Results and Discussion

### 2.1 3D Ink‐Extrusion Printing

**Figure 1:**
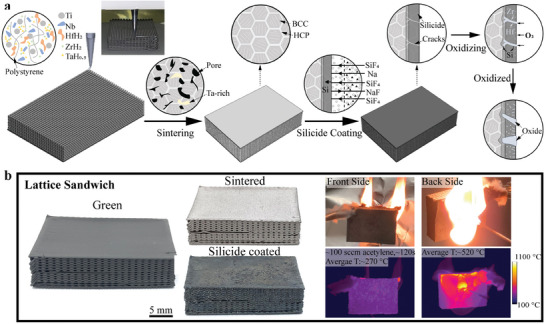
a) Schematic illustration of 3D ink extrusion printing, sintering, and silicide coating of a lattice‐core sandwich; oxidation mechanisms are also shown. b) Photographs of 3D ink extrusion printed, sintered, and silicide‐coated lattice‐core sandwich. In (b), the lattice‐core sandwich is subjected to an acetylene flame to demonstrate its performance for thermal insulation; thermal imaging shows a wide difference in temperature between the two faces of the sandwich.

The 3D ink extrusion printing, sintering, and silicide coating processes are shown in **Figure** **1**a. The ≈410 µm filaments containing metallic/hydride powders are printed layer by layer to produce complex parts, including a lattice sandwich structure, as shown in Figure 1b. When the filaments are extruded, the solvent (dichloromethane, DCM) evaporates near‐instantaneously, and the binder (polystyrene, PS) precipitates, leading to a quick increase in the strength of filaments that helps the structure to self‐support. After sintering, these samples are nearly fully densified and show neither cracking nor warpage. After a subsequent silicide coating, the oxidation resistance of the 3D‐printed part is very significantly improved, which is a key step to making 3D‐printed RHEA structures useful at high temperatures.

The thermal conductivity of sintered HfNbTaTiZr (Figure S1, Supporting Information) is 10–17 (W (m K)^−1^) between 50 and 900 °C, which is lower than the thermal conductivity of pure Hf, Nb, Ta, Ti, and Zr. Low thermal conductivity has been reported in a high‐entropy Al_0.3_CoCrFeNi alloy, where the distorted lattice structure and many lattice defects (e.g., grain boundaries and dislocations) depress the electron/phonon propagation.^[^ ^32^ ^]^ This low thermal conductivity of HfNbTaTiZr makes it desirable for thermal insulation applications. Furthermore, 3D ink‐extrusion printing can fabricate complex porous architectures which also improves thermal insulation. Therefore, here, an oxygen‐resistant RHEA lattice‐core sandwich structure is fabricated by 3D‐ink extrusion printing and silicide coating. The RHEA sandwich structure is then tested with a pure acetylene flame (≈100 SCCM acetylene burning in the air) to demonstrate its thermal insulation performance (Figure S2, Supporting Information). After 120 s heating, the highest local temperature on the hot side is 1100 °C with an average temperature of ≈520 °C, while the average temperature on the cold side is ≈270 °C (Figure 1d). This demonstrates our method's robustness and flexibility for rapid prototyping of RHEA for high‐temperature, ductile, low‐mass insulating structures. Active cooling by a fluid or filling with a low‐conductivity aerogel to prevent cavity radiation (Figure S3, Supporting Information) within the lattice core could further be implemented.

### 2.2 Sintering and Microstructure

Previously, hydride powders have been used for sintering titanium because the dehydrogenation process is reported to remove the oxide layer on the powder surface by releasing H_2_O (steam) which facilitates the subsequent sintering process.^[^ ^33^, ^34^, ^35^ ^]^ Though the reduction of TiO_2_ by H_2_ gas is thermodynamically unfavorable, prior researchers suggest that atomic H in hydride powder will have a negative free energy change after it reacts with O in TiO, TiO_2_, and Ti_2_O_3_ to form steam.^[^ ^33^, ^34^, ^36^ ^]^ In addition, brittle hydride powder can easily provide fine particle sizes by milling, which is beneficial for sintering kinetics.

**Figure 2:**
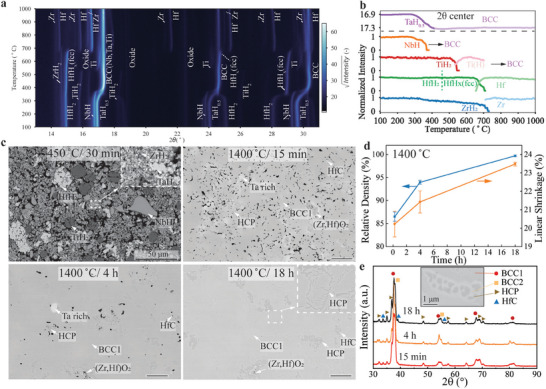
a) Stacked X‐ray spectra acquired during *in‐situ* diffraction of a blend of TiH_2_‐ZrH_2_‐HfH_2_‐NbH‐TaH_0.5_ powders upon heating in vacuum from 100 to 1000 °C. b) corresponding normalized diffraction peak intensity, as a function of temperature, for observed phases: TaH_0.5_, β‐BCC phase, NbH, TiH_2_, Ti‐β (H‐rich), ZrH_2_, Zr, HfH_2_, and Hf. c–e) ink ingot samples vacuum‐sintered for various times (0.25, 4, and 18 h) at 1400 °C: c) SEM‐BSE micrographs of cross‐sections d) relative density e) X‐ray diffractograms showing the formation of two β‐BCC (1 and 2) and one α‐HCP metallic phases, as well as HfC.

The dehydrogenation mechanisms of a blend of TiH_2_‐ZrH_2_‐HfH_2_‐NbH‐TaH_0.5_ powder are first studied by in‐situ X‐ray diffraction. The hydride powder blend is obtained after hydrogenating of an extruded ink at 730 °C for 2 h, using pure hydrogen. As shown in **Figure** **2**a,b, subsequent dehydrogenation of the powder blend under vacuum takes place between 300 and 750 °C. First, TaH_0.5_ and NbH start to decompose at ≈290 °C. The TaH_0.5_ peaks continuously shift to higher diffraction angles until they merge with the β‐BCC (Body‐Centered Cubic) peaks at ≈410 °C, indicating a continuous loss of H, consistent with the Ta‐H phase diagram^[^ ^37^ ^]^ showing a Ta(H) BCC solid solution above ≈50 °C for H/Ta = 0–0.8. Decomposition of NbH ends at ≈380 °C. Subsequently, the Group IV hydrides decompose fully at i) ≈545 °C for TiH_2_ and at ≈700 °C for β‐Ti (H‐rich), ii) at ≈460 °C for HfH_2_, and at ≈700 °C for HfH_x_, and iii) at ≈720 °C for ZrH_2_. The sequence of the hydrides decomposition closely follows the sequence of hydride formation enthalpy values for Ta (−34 kJ mol^−1^), Nb (−40 kJ mol^−1^), Hf (−64 kJ mol^−1^), Ti (−67 kJ mol^−1^), and Zr (−79 kJ mol^−1^).^[^ ^38^ ^]^ After dehydrogenation of β‐Ti (H‐rich) at 700 °C, α‐Ti is expected because the transition temperature between the α‐HCP (Hexagonal Close‐Packed) phase and β‐BCC phase in pure Ti is at 882 °C. The absence of α‐Ti indicates that Nb and Ta (which are β‐stabilizer for Ti) are already alloyed with the nascent Ti, thus decreasing the allotropic temperature. As a result, peaks of metallic Ti, Nb, and Ta merge together to form the β‐BCC phase. On the other hand, α‐Zr and α‐Hf peaks appear after dehydrogenation and remain visible up to the maximum test temperature of 1000 °C. As Zr has an α‐β transition temperature of 863 °C which is lower than 1000 °C. The absence of β Zr above 863 °C indicates that it alloys probably with Hf, which is α‐stabilizer and has an α‐to‐β transition temperature of 1743 °C. Furthermore, peaks for (Zr,Hf)O_2_ are present at the beginning of the heating, suggesting that some oxidation occurred during the prior hydrogenation process at 730 °C for 2 h. In order to avoid this oxidation, further specimens reported below were heated under hydrogen to a lower temperature of 450 °C for a shorter time of 30 min for the de‐binding process (Figure S4, Supporting Information).

The evolution of microstructure for ink‐cast ingot samples after heating to 1400 °C and vacuum‐sintering for 0.25, 4, and 18 h are shown in Figure 2c. After the initial de‐binding under hydrogen (450 °C/ 30 min), the sample shows a loosely‐packed powder blend consisting of TiH_2_, ZrH_2_, HfH_2_, NbH, and TaH_0.5_. After sintering at 1400 °C for the shortest time (15 min), the microstructure remains porous and consists of three metallic phases – (Ti,Nb,Ta)‐rich, (Zr,Hf)‐rich, and Ta‐rich – (Zr,Hf)O_2_ and HfC particles. As shown in the XRD patterns in Figure 2e, the (Ti,Nb,Ta)‐rich phase corresponds to the β‐BCC1 phase, and the (Hf, Zr)‐rich phase corresponds to the α‐HCP phase. The undiffused Ta‐rich phases are consistent with the occurrence of large agglomerates of Ta powders and their low ability to homogenize with the other metallic phases, due to the low Ta diffusivity. The formation of (Zr,Hf)O_2_, driven by oxygen contamination during sintering, is expected, given that Hf and Zr oxides are more stable than the oxides of Ti, Ta, and Nb, and thus form preferentially.^[^ ^39^ ^]^ The formation of HfC, caused by carbon contamination during debinding, is anticipated, given that HfC is more stable than the carbide of Zr, Ta, Nb, and Ti, and thus forms preferentially.^[^ ^40^ ^]^ After sintering for a longer time (4 h), the microstructure is further densified, and the Ta‐rich phases are smaller, as Ta diffused into the surrounding metallic phases for a longer time. Finally, after sintering for the longest time (18 h), the microstructure is nearly fully densified, consistent with a high relative density of 99.7 ± 0.2% and a large linear shrinkage of 23.5 ± 0.1% (Figure 2d). A second β‐BCC phase (BCC2) is visible in the XRD spectra and the SEM‐BSE micrographs in Figure 2e. This β‐BCC2 phase forms around the α‐HCP phase; it has a composition close to the β‐BCC1 phase, but is slightly enriched in Ta and Nb and slightly depleted in Ti, Zr, and Hf, as shown in **Table** **1**.

**Table 1:** Composition (at.%) from EDS for each phase shown in Figure 2e, normalized after excluding oxygen and carbon.

Phase	Hf	Ta	Nb	Ti	Zr
β‐BCC‐1	17.8	21.0	20.3	25.1	15.8
β‐BCC‐2	15.8	24.7	21.6	22.8	15.1
α‐HCP	32.1	7.9	4.6	14.9	40.6

**Figure 3:**
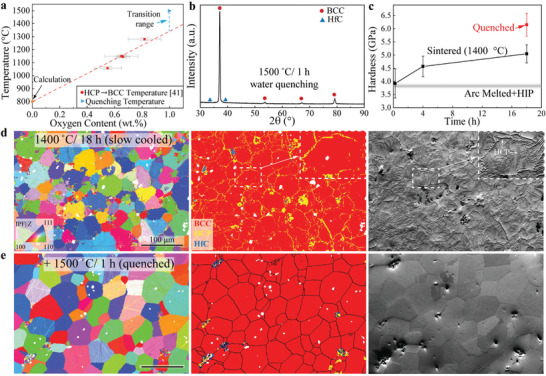
a) The α‐to‐β phase transition temperature as a function of oxygen content.^[^ ^41^ ^]^ The transition temperature at 0 wt.% oxygen is calculated by Thermo‐Calc. The homogenization temperature (1500 °C/ water quenched) of our sintered samples (1400 °C/ 18 h) is added to show that their transition temperature should be well below 1500 °C given their measured oxygen content of 0.996 wt.%. b) XRD spectrum after water quenching from a sample after 1 h homogenization at 1500 °C, showing the presence of a metastable β‐BCC phase with residual,HfC. c) Hardness of sintered samples as a function of sintering time at 1400 °C and hardness of the water‐quenched sample. The hardness of an arc‐melted, hot isostatic pressed sample is added for comparison.^[^ ^42^ ^]^ Inverse Pole Figure (IPF) map, phase map, and forward‐scattered electron (FSE) micrograph from EBSD for (d) a sintered sample (1400 °C / 18 h) after slow cooling (e) a sintered sample (1400 °C / 18 h) after water quenching from homogenization at 1500 °C/ 1 h.

Arc‐melted equiatomic HfNbTaTiZr is well known to display a metastable single β‐BCC phase, which is the microstructure that has been so far studied and tested in the literature.^[^ ^23^, ^25^, ^43^ ^]^ However, a multiphase (β‐BCC1 + β‐BCC2 + α‐HCP) microstructure is obtained in our specimens, after a very different processing route, i.e., sintering at 1400 °C for 18 h followed by slow cooling (5 °C min^−1^). To understand the reason for the formation of the current microstructure, a 1 h homogenization at 1500 °C followed by water quenching is performed to simulate the cooling rate of arc melting. Previously, Jaroslav et al. performed water quenching from 1200 °C/ 1 h for their sintered samples and still found much residual α‐HCP phase.^[^ ^41^ ^]^ They attribute it to dissolved oxygen accumulated during sintering, which increases the α‐to‐β transition temperature as oxygen is an α‐HCP stabilizer. Thus, their samples' transition temperature is higher than 1200 °C.^[^ ^41^ ^]^ Their transition temperature as measured by DSC as a function of oxygen content is summarized in **Figure** **3**a. The calculated transition temperature from Therma‐Calc (package: TCHEA5) is added to give a guideline for higher oxygen content. The oxygen content of our sintered samples is 0.996 wt.%, as shown in Table S2 (Supporting Information). Based on the data shown in Figure 3a, we perform quenching from 1500 °C to ensure the α‐to‐β transition. A metastable single β‐BCC phase (with residual HfC) is obtained after quenching, as shown in the XRD spectrum of Figure 3b. This indicates that the α‐to‐β transition temperature should be between 1280 °C (extrapolated from the guideline) and 1500 °C at our oxygen concentration of 0.996 wt.%. A slightly higher content of carbon content (0.102 wt.%) shown in Table S2 (Supporting Information) can also contribute to a higher α‐to‐β transition temperature,^[^ ^44^ ^]^ making the transition temperature higher than 1280 °C. The hardness of the sintered samples as a function of sintering time at 1400 °C and the hardness of the water‐quenched sample are shown in Figure 3c. The hardness of the sample increases with sintering time mainly due to the densification of the alloy. After quenching, the metastable single β‐BCC sample shows a higher hardness than the multiphase, sintered sample, for two possible reasons: a) Hf and Zr diffused into the β‐BCC matrix and contribute to substitutional solid‐solution strengthening as Hf (0.225 nm) and Zr (0.160 nm) have the largest and smallest atomic radius within these five elements (Ti: 0.187 nm, Nb:0.207 nm, Ta: 0.220 nm); b) Hf and Zr in the α‐HCP phase have a more negative free energy formation of oxide formation^[^ ^39^ ^]^ and thus absorb more oxygen during sintering. When Hf and Zr diffuse into the β‐BCC matrix, the oxygen content in the β‐BCC phase may increase, and thus contribute to interstitial solid‐solution strengthening. In addition, both the sample sintered at 1400 °C and the quenched sample show a higher hardness than the arc‐melted and hot isostatic pressed (HIP) sample,^[^ ^42^ ^]^ which we expect to be due to the strong interstitial strengthening effect from oxygen and carbon.

An Inverse Pole Figure (IPF) map, a phase map, and a forward‐scattered electron (FSE) micrograph are shown in Figure 3d for the sintered sample (1400 °C / 18 h) and quenched samples (after homogenization at 1500 °C / 1 h). The sintered sample has equiaxed β‐BCC grains with a grain size of 28 ± 14 µm. The α‐HCP phase segregates around the β‐BCC grain boundary and also appears inside the BCC grains. After quenching, the α‐HCP phase disappears, and a metastable β‐BCC phase with a grain size of 33 ± 19 µm is formed (Figure 3e), in accordance with XRD results. Due to the similar lattice parameters of the β‐BCC1 and β‐BCC2 phases, the IPF and phase maps cannot identify them separately. However, a single β‐BCC phase is shown in the XRD pattern in Figure 3b. These results suggest that the α‐HCP phase and dual β‐BCC phases are formed during slow cooling after sintering and not by insufficient diffusion of each element during sintering (Figure S5, Supporting Information). The HfC is also indexed in the phase map, consistent with XRD results in Figure 3b.

### 2.3 Mechanical Properties

**Figure 4:**
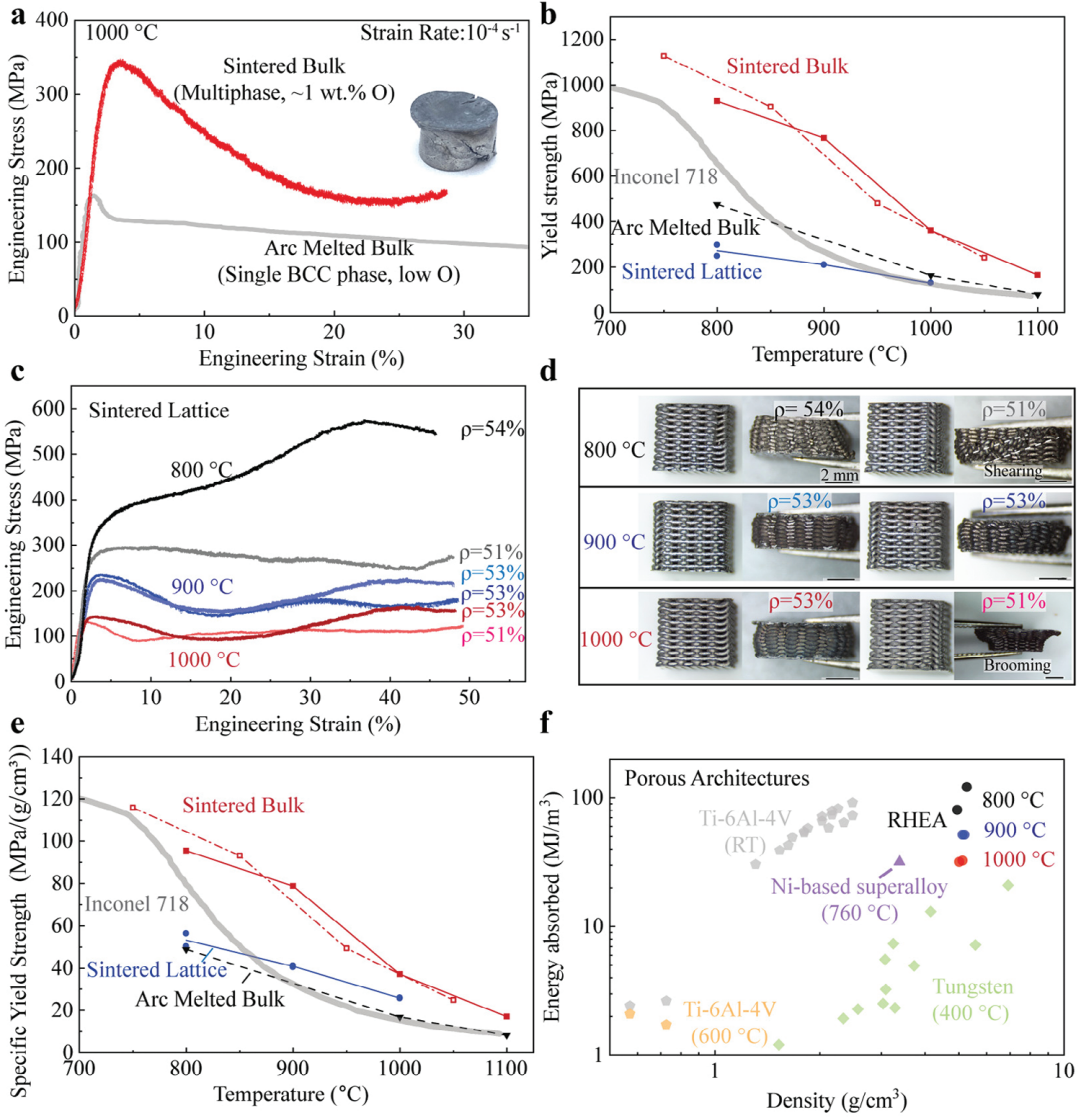
a) Compressive stress–strain curves, measured at 1000 °C, for sintered bulk HfNbTaTiZr specimen (current study) and arc‐melted bulk HfNbTaTiZr specimen.^[^ ^25^ ^]^ b) Temperature dependence of yield strengths for sintered bulk HfNbTaTiZr (two samples: open rectangle and solid rectangle) and sintered lattices. The yield strengths of Inconel 718 and arc‐melted bulk HfNbTaTiZr are added for comparison (from Refs. [25, 45, 46]). c) Compressive stress‐strain curves of HfNbTaTiZr lattices at 800, 900, and 1000 °C at an initial strain rate of 10^−4^ s^−1^. d) Photographs showing side views of HfNbTaTiZr lattices before and after deformation. e) Specific yield strength of sintered HfNbTaTiZr (bulk and lattices) as a function of temperature. f) Energy absorption as a function of density for porous architectures from current HfNbTaTiZr, Ti‐6Al‐4 V,^[^ ^47^, ^48^ ^]^ tungsten,^[^ ^49^ ^]^ and Ni‐based superalloy,^[^ ^50^ ^]^ at various temperatures with an upper integration strain of 0.3.

As the single β‐BCC microstructure is metastable, it is expected to decompose during hot deformation below the transition temperature.^[^ ^51^ ^]^ Therefore, only the sintered samples with the stable multiphase microstructure are mechanically tested. The compressive behavior of sintered HfNbTaTiZr lattice and bulk samples is described in **Figure** **4**. Figure 4a,b shows that the yield strength is much higher for sintered HfNbTaTiZr bulk than for arc‐melted HfNbTaTiZr reported in Refs. [25, 45] between 750 to 1100 °C. A relatively high content of oxygen (0.996 wt.% or 6.7 at.%) and carbon (0.102 wt.% or 0.9 at.%) are measured in the as‐sintered sample. Yuan et al. studied the interstitial strengthening effect from O and C on HfNbTiZr alloy.^[^ ^52^ ^]^ They found that the increase of yield strength ∆σ at room temperature is: ∆σ = 261 MPa ×[O at.%]^1/2^ and 100 MPa × [C at.%]^1/2^ after fitting their experimental data with the Fleischer model in BCC metals.^[^ ^52^ ^]^ This indicates that oxygen interstitial strengthening may significantly increase yield strength in the current HfNbTaTiZr samples: using the above equations provides strengthening contributions of 676 and 95 MPa, respectively. Also, delaying recrystallization by interstitial oxygen^[^ ^53^ ^]^ can also contribute to a higher strength at high temperatures for alloys with fine grain sizes. However, the microstructure here has relatively coarse grains (≈28 ± 14 µm), indicating that Hall‐Petch strengthening is not the dominant strengthening mechanism. In fact, the sintered HfNbTaTiZr samples show an even higher yield strength than Inconel 718 between 750 and 1100 °C. The stress drop after yielding at 1000 °C shown in Figure 4a may be due to dynamic recovery and recrystallization which were reported previously for arc melted HfNbTaTiZr.^[^ ^23^, ^25^ ^]^ In addition, cracks forming along shearing bands, observed on the heavily deformed sample (inset in Figure 4a), may also have contributed to the softening of the flow stress.

In addition to bulk samples, 3D‐printed microlattices were mechanically tested. As shown in Figure 4c,d, the sintered lattice can be compressed without fracture up to 50% between 800 and 1000 °C. Two lattices tested at 800 °C show different yield strengths and subsequent work‐hardening behaviors. This is because one lattice (ρ = 51%) shows a shearing behavior (Figure 4d) during deformation with deformation concentrated at the shearing band. This results in a lower yield strength and lower work hardening. Both samples compressed at 900 °C have similar compression stress‐strain curves, with both lattices showing barreling behavior. A decrease of stress after yielding is observed beginning at 900 °C, indicating that dynamic recovery and dynamic recrystallization may become dominant at this temperature, in comparison with the sample compressed at 800 °C. After the initial stress drop, the progressive densification of lattices leads to higher stress and competes with the subsequent softening of the flow stress. Therefore, a long stress plateau is present in compression curves. After deformation in uniaxial compression at 900 °C, the lattices show lateral expansion, where horizontal struts are plastically deformed in tension without cracking or fracturing, indicating high tensile ductility at high temperatures (Figure S9, Supporting Information). The yield strength of lattices tested at 1000 °C decreases further, with flow stress also decreasing as compared to the 900 °C tests. However, the sample with a relative density of ρ = 51% shows a brooming behavior (Figure 4d) that may explain its larger stress drop after yielding.

The specific yield strengths of sintered HfNbTaTiZr lattices and bulk samples are shown in Figure 4e. The sintered bulk samples show a higher specific yield strength than Inconel 718 and arc melted samples between 750 and 1100 °C. The sintered lattices have a higher specific yield strength than Inconel 718 above 900 °C. This makes the current lattices promising for high‐temperature applications where light weight is needed. Finally, the energy absorption (upper integration strain: 0.3) of these lattices is summarized in Figure 4f, which shows that the RHEA lattices can absorb large amounts of energy via plastic deformation at 800–1000 °C; for comparison, data for tungsten (400 °C), Ti‐6Al‐4 V (600 °C), and nickel‐based superalloy (760 °C) porous architectures are also given.

### 2.4 Oxidation Resistance

**Figure 5:**
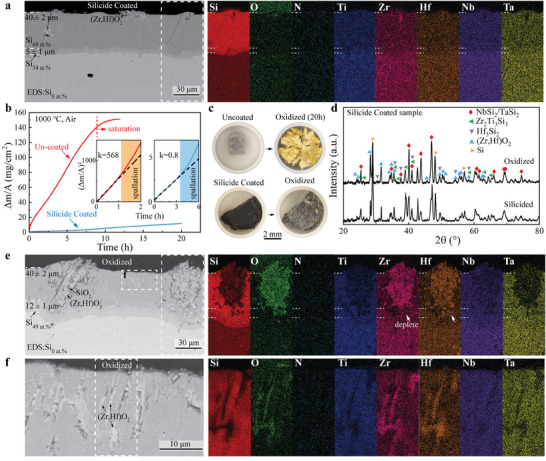
a) SEM‐BSE micrographs of cross‐sections of the silicide‐coated HfNbTaTiZr bulk sample after the pack cementation process. EDS maps of the region identified by a white dotted box show the distribution of the various elements (Si, O, N, Ti, Zr, Hf, Nb, and Ta). b) Mass gain (per area, Δm/A) for the uncoated and silicide‐coated samples as a function of time at 1000 °C under flowing air. The insets show the square of mass gain per area (Δm/A)^2^ as a function of time, where the linear‐increase region (steady–state oxidation) is fitted to get the parabolic rate constant k. c) Photographs of the uncoated and silicide‐coated samples before and after oxidation at 1000 °C for 20 h. d) XRD spectra from the surface of the silicide‐coated samples before and after oxidation. e) SEM‐BSE micrographs and EDS maps of cross‐sections of the silicide‐coated sample after oxidation at 1000 °C for 20 h. f) Enlarged SEM‐BSE micrographs and EDS maps of the region identified by a white dotted box in (e).

Oxidation resistance is crucial for high‐temperature applications. Here, we use a pack cementation process to create a conformal silicide coating on the sintered HfNbTaTiZr samples to improve oxygen resistance. During pack cementation at 950 °C for 24 h, the fluoride activator reacts with the Si source to form gaseous SiF_4_, which deposits Si on the sample driven by the chemical potential gradient in the pack. Because Si is the faster diffusing element in the silicide of Ti,^[^ ^54^ ^]^ Zr,^[^ ^55^ ^]^ Hf,^[^ ^55^ ^]^ Nb,^[^ ^56^ ^]^ and Ta,^[^ ^57^ ^]^ inward diffusion of Si occurs from the surface of the sample, thus forming two distinct silicide layers as shown in **Figure** **5**a. The first layer, with a thickness of 40 ± 2 µm, has a high silicon content (69 at.%) which roughly corresponds to MSi_2_ (M = Ti, Zr, Hf, Nb, and Ta). The second, layer is much thinner (5 ± 1 µm) and exhibits a silicon content of 54 at.%, which roughly corresponds to MSi. The XRD spectra shown in Figure 5d confirm the formation of crystal structures of NbSi_2_/TaSi_2_, Zr_2_Ti_3_Si_3_, and Hf_3_Si_2_ phases, according to existing PDF cards (the complete indexing of further minor XRD peaks is not carried out). The silicon signal below the silicide layers is below the EDS detection limit. In addition, cracks are found in the silicide layer and these cracks end at the interface between the silicide and the metallic substrate. These cracks may be created during cooling because of the mismatch of coefficients of thermal expansion between the silicide layer and substrate, as observed in aluminide coatings.^[^ ^28^ ^]^ Occasional (Zr,Hf)O_2_ particles are observed in the silicide layer, which may have pre‐existed at the surface of the alloy or may have formed during the pack cementation process.

After silicide coating, the uncoated and coated specimens were tested by Thermogravimetric analysis (TGA) at 1000 °C under airflow, with oxide formation responsible for the observed mass gain per unit area shown to increase nearly linearly with time in Figure 5b. The mass gain of the silicide‐coated sample is much lower than that of the uncoated sample, confirming that the silicide coating inhibits the oxidation of HfNbTaTiZr RHEA effectively. Steady–state oxidation of metals and alloys often follows parabolic rate kinetics:

(1) $${\left(\Delta m/A\right)}^{2}=kt$$

where Δm is the mass gain, *A* is the surface area, *k* is the parabolic rate constant, and *t* is the time. The parabolic rate constant *k* is directly related to the self‐diffusion coefficient of oxygen (or metallic elements) in the oxide scale and thus fully describes the oxidation kinetics during the early stage growth of the oxide layer.^[^ ^58^, ^59^ ^]^ In Figure 5b, for the first 1–3 h of oxidation, the specimens appear to follow the parabolic law, and parabolic rate constants can be determined for the uncoated sample (*k* = 570 mg^2^ cm^−4^ h^−1^), which is ≈700 times higher than that of silicide‐coated sample (*k* = 0.80 mg^2^ cm^−4^ h^−1^), indicating much higher oxidation resistance for the silicide‐coated samples. After 3 h oxidation, the mass gain of the silicide‐coated sample is Δ*m*/*A* = 1.6 mg cm^−2^, which is similar to the value of 1.4 mg cm^−2^ for Inconel 718 at 1000 °C in air.^[^ ^60^ ^]^ At the later stage of oxidation, both RHEA samples show a nearly linear increase of Δ*m*/*A* with time, which usually corresponds to fracture or spallation of the oxide scale. The increase of Δ*m*/*A* for the uncoated sample starts to slow after 9 h suggesting that the sample begins to saturate with oxygen internally, leading to a smaller concentration difference of metal/oxygen across the scale. After 10 h oxidation, the uncoated sample has a Δ*m*/*A* of 148 mg cm^−2^, which is ≈30 times higher than 5.2 mg cm^−2^ for the silicide‐coated sample. This shows that the silicide layer is still quite protective, well beyond the early parabolic oxidation stage. The mass of the uncoated sample becomes stable after 12 h due to the completion of oxidation.

Photographs of the uncoated and silicide‐coated samples, before and after the 20 h oxidation tests, are shown in Figure 5c. The uncoated sample is pulverized by oxidation‐induced volume expansion and shows a yellow color, which is similar to the pesting behavior reported for Hf_0.5_Nb_0.5_Ta_0.5_Ti_1.5_Zr at 1000 °C.^[^ ^27^ ^]^ The silicide‐coated sample maintains its shape and shows white and yellow regions on its surface, indicating some oxidation.

Cross‐sections of the silicide‐coated sample after oxidation at 1000 °C for 20 h are shown in Figure 5e,f. The SEM‐BSE and EDS maps show that pre‐existing cracks are filled with (Zr,Hf)O_2_ after oxidation, consistent with Zr and Hf having more negative free energy of oxide formation than Si.^[^ ^39^, ^61^ ^]^ Thus, Zr and Hf within the silicide coating react with oxygen to form the oxide, while Si is released and diffuses deeper in the specimen, increasing the depth of the coating. This is illustrated in Figure 5e, showing a large (Zr,Hf)O_2_ inclusion (probably formed at a crack), below which a deeper incursion of the silicide layer is visible. Thus, the formation of (Zr,Hf)O_2_ filling cracks and the growth of the silicide layer underneath it both prevent the run‐away oxidation of the surface via spalling. This self‐healing mechanism ensures good oxygen resistance even with the existence of cracks in the brittle silicide layer. In addition, some SiO_2_ particles are observed within the large (Zr,Hf)O_2_ inclusion, which is caused by insufficient Hf and Zr around the big (Zr,Hf)O_2_ particles as shown in the depletion zone of the Hf and Zr EDS maps. These SiO_2_ particles can be helpful in relieving the stress between oxide grains by viscous flow.^[^ ^62^, ^63^ ^]^ Figure 5f is the enlarged SEM‐BSE micrograph showing a region of the oxidized surface without pre‐existing cracks. Lamellar (Zr,Hf)O_2_ inclusions are visible within the coating. The lamellar structure may be inherited from the morphology of the α‐HCP (Zr,Hf‐rich) phase, shown in Figure 2c.

## 3 Conclusions 

This study demonstrates the synthesis of equiatomic HfNbTaTiZr refractory high entropy alloy (RHEA) into complex shapes via printing of an ink containing precursor metallic or hydride powders, which is then sintered and homogenized. The main conclusions are:

1. For a blend of hydride powders – TiH_2_, ZrH_2_, HfH_2_, NbH, TaH_0.5_ – heated from 100 to 1000 °C, in situ X‐ray diffraction reveals that the hydrides decompose sequentially starting with Ta and Nb, to Ti, Hf, and eventually Zr. A nearly fully densified multiphase (β‐BCC1+ β‐BCC2+α‐HCP) alloy is achieved after sintering at 1400 °C for 18 h, followed by slow cooling. A metastable single β‐BCC microstructure is obtained after quenching.
2. Sintered bulk and micro‐lattices RHEA samples are mechanically tested at elevated temperatures. The sintered bulk samples show higher yield strength than arc‐melted samples, consistent with the interstitial strengthening of oxygen (≈1.0 wt.%), between 750 and 1100 °C. Sintered RHEA microlattices are ductile, with high energy absorption, under compression at high temperatures, and show a higher specific yield strength than Inconel 718 above 900 °C.
3. Sintered RHEA is silicide‐coated via the pack cementation method, leading to the formation of two silicide layers with thicknesses of 40 and 5 µm, and with Si contents of 69 and 54 at.%, respectively. During the oxidation test at 1000 °C, the parabolic rate constant of the silicide‐coated alloy is almost a thousand‐fold lower than that of the uncoated alloy, showing much lower oxidation kinetics. This is explained by the formation of (Zr,Hf)O_2_ filling cracks within the silicide layer and protecting the underlying alloy by expelling Si under the cracks.
4. A HfNbTaTiZr RHEA item with a complex shape –lattice‐core sandwich structure – is fabricated by 3D ink extrusion printing, sintering, and silicide coating.

## 4 Experimental Section

### Ink Preparation and 3D Printing

Inks for 3D printing were prepared by mixing and suspending five powders – Ti (mass: 2.96 g, purity: ≥99%, powder size: <25 µm, supplier: AP&C), ZrH_2_ (5.66 g, 99%, 2 µm, US Research Nanomaterials, Inc), HfH_2_ (10.95 g, >99.9%, 1–10 µm, NANOCHEMAZONE), Nb (5.75 g, 99.8%, 1–5 µm, Thermo Scientific), TaH_0.5_ (11.01 g, 99.9%, 2 µm, Thermo Scientific) powders – in dichloromethane (40 mL, DCM, Sigma–Aldrich), with the following additions: i) polystyrene (1.67 g, PS, Mw = 350 k, Sigma–Aldrich) as binder, ii) dibutyl phthalate (0.4 g, DBP, Sigma–Aldrich) as plasticizer, iii) ethylene glycol butyl ether (2.4 g, EGBE, Sigma–Aldrich) as surfactant. The Ti and Nb powder are each purposely slightly over‐stoichiometric (as 20.2 at.%) in the powder blend, to compensate for the possible loss during ball milling (as ductile powders often stick to the balls). The oxygen content of each powder is shown in Table S1 (Supporting Information) and spans 0.29–0.83 wt.%. Roller milling was carried out in high‐density polyethylene (HDPE, 120 mL) bottle for 12 h to mix the full wet ink with zirconia balls (ball to powder ratio: 1:1). The viscosity of the inks was adjusted by DCM evaporation at 50 °C to get a printable ink (the rheological properties of the PS‐DBP‐DCM ink system have been studied in detail in Ref. [64]). Extrusion printing was performed with a 3D‐Bioplotter (EnvisionTEC, Germany) with conical 410 µm plastic nozzles (Nordson EFD). The 9 × 9 × 9 mm^3^ lattices were printed with a layer height of 320 µm, a horizontal spacing of 0.8 mm, and a layer rotation angle of 90°. Furthermore, three complex objects were printed. First, a sandwich structure (25 × 35 × 10 mm^3^_)_ with two dense skins (single printed layer with a horizontal spacing of 0.35 mm) is sandwiching the same lattice structures described above. Finally, inks with lower viscosity than above (with a reduced level of DCM evaporation) were also extruded into a rectangular mold (30 × 30 × 5 mm^3^), forming a uniform, bulk ingot. After drying, these ink‐cast ingots were cut with a razor blade into cuboids (approximate size: (7‐13) × (5‐7) × 5 mm^3^) which were used to explore the sintering and siliconizing processes.

### Heat Treatment and Silicide Coating

First, the printed samples were exposed to flowing hydrogen (99.999% pure) for de‐binding. Two steps were included in the de‐binding stage: i) evaporation of residual DCM solvent and EGBE at 150 °C for 30 min, and ii) decomposition of the PS binder polymer at 450 °C for 30 min. The heating and cooling rates are 10 °C min^−1^. Second, sintering was performed at 1400 °C, for times spanning between 15 min and 18 h, in a vacuum tube furnace with an initial vacuum level of < 3 × 10^−6^ torr. A zirconium foil was used to cover the crucible containing the samples and titanium sponges were placed in the crucible to act as an oxygen getter during sintering. Some sintered samples were then wrapped with Mo foil, sealed in an evacuated quartz tube, heated at 1500 °C for 1 h for homogenization, and then water quenched, to study phase transformations in the alloy. To determine the intrinsic mechanical properties of the sintered alloy, bulk cylinder samples with an aspect ratio of 2:1 (load‐unload compression tests) and 1.2:1 (compression test at 1000 °C) are prepared by cold‐pressing and vacuum sintering of dry powder mixtures which were collected from the same inks described above, after a de‐binding step.

A halide‐activated pack cementation process was used to deposit a silicide coating on the sintered samples, following Ref. [65] A powder pack was prepared by roller mixing for ≈1 h, consisting of i) 24.6 wt.% pure Si (99.9985%, 1–20 µm, Alfa Aesar) as Si source, ii) 73.8 wt.% alumina (99.95%, Alfa Aesar) as filler, and iii) 1.6 wt.% sodium hexafluoroaluminate (99.4%, <20 um after sieving) as halide activator. The sintered samples were buried in the powder pack, sealed in an evacuated, argon‐backfilled (≈200 mbar), and carbon‐coated quartz tube, and heat‐treated at 950 °C for 24 h under flowing argon, with a heating/cooling rate of 5 °C min^−1^.

### Characterization

In situ X‐ray diffraction was performed on a STADI MP instrument to study the dehydrogenation mechanisms. The measurement was performed with a Mo source on the TiH_2_‐ZrH_2_‐HfH_2_‐NbH‐TaH_0.5_ powder blend (target composition: equiatomic HfNbTaTiZr) lodged in an externally heated quartz capillary (0.3 mm ID). The hydride powder blend was obtained after hydrogenating extruded ink at 730 °C for 2 h under pure hydrogen. The subsequent dehydrogenation experiments were performed under vacuum (≈10^−3^ torr) while increasing temperature from 100 to 1000 °C with diffraction spectra collected at each 5 °C step with 18.54° 2θ angular coverage and 120 s exposure. Data post‐processing and plotting were performed using Python (Anaconda, Continuum Analytics). Perceptually uniform color maps were retrieved from the cmocean package.^[^ ^66^ ^]^ All diffractograms were background‐corrected using asymmetric least‐squares smoothing.^[^ ^67^ ^]^ Each diffraction peak was fit with a Pseudo‐Voight function by using the lmfit package.^[^ ^68^ ^]^

Linear shrinkages of sintered samples were measured from photographs (taken at low magnification with a stereo microscope) before and after sintering. Metallographic characterization was done on polished cross‐sections of ink‐extruded cuboid samples. Samples were cold‐mounted in epoxy resin, ground with SiC grinding papers, polished with diamond suspensions (3 and 1 µm) and colloidal silica (0.02 µm), and coated with 8 nm Os. Scanning electron microscopy (SEM) and electron backscatter diffraction (EBSD) were performed on a Quanta 650 instrument with an Oxford Symmetry 2 detector. Relative densities of sintered samples were measured from partial cross‐sections, from at least five optical micrographs (MA200 Automated Microscope). Ex‐situ X‐ray diffraction was performed on polished cross‐sections of sintered and quenched samples, and on the surface of silicided samples, using a Smartlab 3 kW Gen2 instrument.

Vickers microhardness was averaged from at least ten indentations measured on a Wilson VH3100 automated micro‐hardness tester, by applying a 1.96 N force for 10 s. High‐temperature compression tests were carried out at an initial strain rate of 1 × 10^−4^ s^−1^ on an MTS‐5 servo‐hydraulic tester with a furnace using flowing argon (99.999% pure). To ensure uniaxial compression, the top and bottom surfaces of sintered samples were ground flat and parallel before the compression test. Then, the sintered lattices (≈5.6 × 5.6 × 5.6 mm^3^) and bulk cylindrical samples (D:5.7 mm, H:11.7 mm) were placed between boron‐nitride‐coated platens and wrapped with Ti foils as oxygen getter. Yield strengths of two bulk cylindrical samples were measured between 800 and 1100 °C and between 750 and 1050 °C, respectively. After yielding (plastic strain: ≈0.2%), the load was removed, and the temperature was increased by ≈100 °C to the next temperature. The subsequent load‐yield‐unload measurement took place after the temperature was stabilized, and this procedure was repeated until the highest temperature was reached.

Thermogravimetric analysis (TGA) was carried out on Mettler Toledo TGA/DSC 3+ instrument. The sintered specimens – uncoated (74 mg, cuboid, 1.8 × 2.2 × 2.1 mm) and silicide‐coated (112 mg, near half disk, R: 3.0 mm, H: 0.9 mm, surface area calculated from optical images) – were placed in alumina crucibles and mass gain was measured at 1000 °C for 20 h under dry air (50 mL min^−1^ flow rate).

Thermal diffusivity (*D_T_*) of the sintered samples between 50 and 900 °C was measured by Laser‐flash analysis (Netzsch LFA 457, Germany). The thermal conductivity was calculated according to *k*  =  ρ*c*_p_ *D*_T_, where ρ is the density (9.72 g cm^−3[^ ^69^ ^]^), and *c*_p_ is the specific heat capacity (approximated by the Dulong–Petit law).

## Conflict of Interest

DCD discloses a financial interest in Metalprinting, Inc. (South Korea) which is active in ink‐based materials printing.

## Author Contributions

D.C.Z. performed all experiments, evaluated the data, and wrote the first draft of the manuscript. Y.C.H. performed preliminary experiments on sintering together with D.C.Z. D.C.D. conceived the experimental plan, discussed all data, and revised the manuscript.

## Supporting information

Supporting Information

## Data Availability

The data that support the findings of this study are available from the corresponding author upon reasonable request.
