# Supplementary material for: Ink‐Extrusion 3D Printing and Silicide Coating of HfNbTaTiZr Refractory High‐Entropy Alloy for Extreme Temperature Applications
Source: Adv Sci (Weinh). 2024 Feb 28;11(17):2309693. doi: 10.1002/advs.202309693 (PMC11077685; doi:10.1002/advs.202309693)
Supplement: Supplementary file 1 — Supporting Information [file ADVS-11-2309693-s001.pdf]

ADVANCED SCIENCE Open Access

Supporting Information

for *Adv. Sci.*, DOI 10.1002/advs.202309693

Ink-Extrusion 3D Printing and Silicide Coating of HfNbTaTiZr Refractory High-Entropy Alloy for Extreme Temperature Applications

Dingchang Zhang, Ya-Chu Hsu and David C. Dunand**

Supplementary Information

Ink-extrusion 3D printing and silicide coating of HfNbTaTiZr refractory high-entropy alloy for extreme temperature applications

Dingchang Zhang*, Ya-Chu Hsu, David C. Dunand*

Department of Materials Science and Engineering, McCormick School of Engineering, Northwestern University, 2220 Campus Drive, Evanston, IL 60208, USA

* Corresponding author: dunand@northwestern.edu; Phone number: +1-847-491-5370
dingchangzhang2020@u.northwestern.com; Phone number: +1-773-865-9150

Supplementary Table S1. Oxygen concentration (as measured from IGF analysis) for HfH_2, $TaH_{0.5}$, Nb, ZrH_2, and Ti powders. The calculated oxygen concentration in the powder blend is also listed.

powder	HfH_2	$TaH_{0.5}$	Nb	Ti	ZrH_2	Blend
O (wt.%)	0.79	0.29	0.59	0.26	0.83	0.50

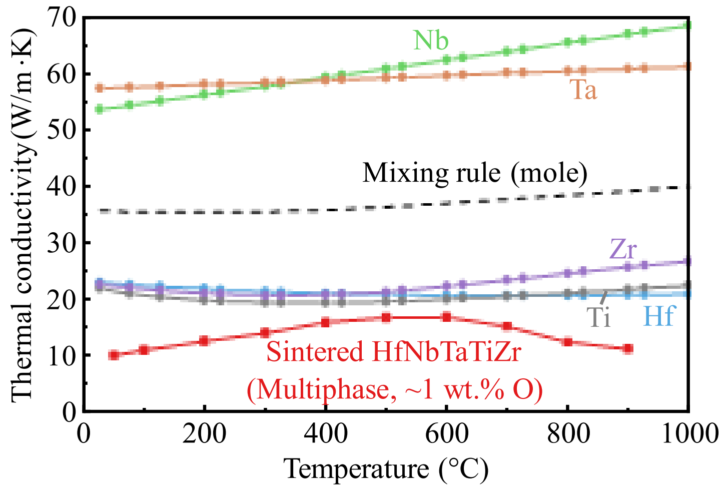

Supplementary Figure S1. Thermal conductivity of HfNbTaTiZr: (red) as measured on sintered (1400 ˚C/18 h) specimen and (black) as calculated via the rule of mixture, using the thermal conductivities of elemental Hf, Nb, Ta, Ti, and Zr [1], based on mole fractions.

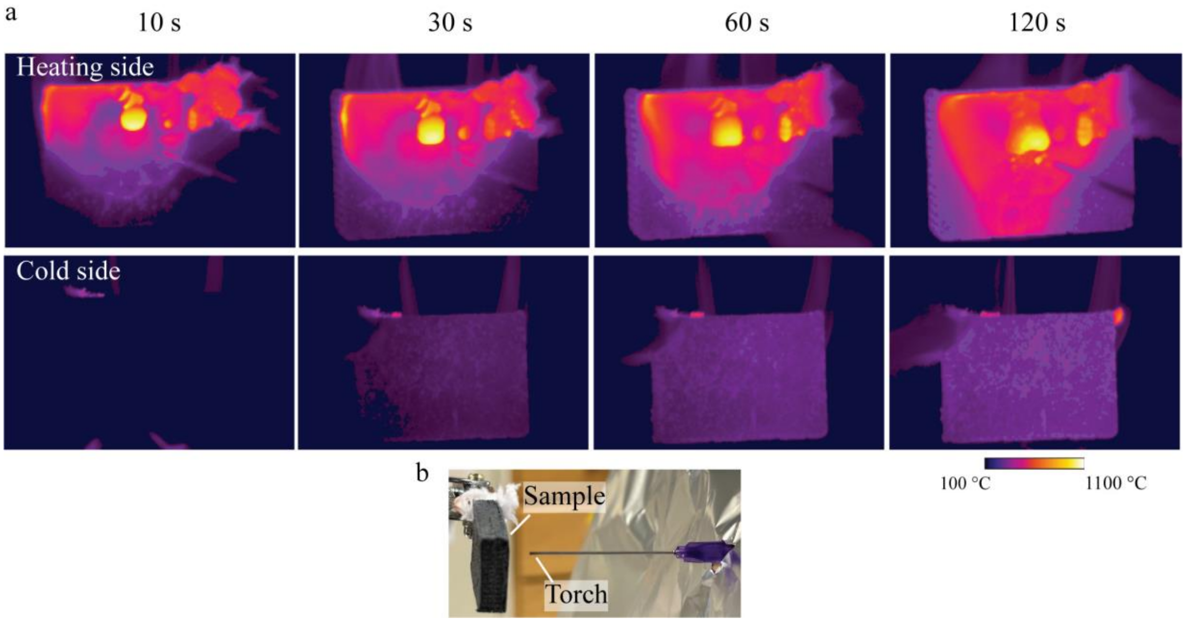

Supplementary Figure S2. (a) Infrared images of silicide-coated lattice-core sandwich structure (~19×27×7 mm^3) during heating by a pure acetylene flame (~ 100 sccm). The heated side and cold side are recorded separately but under the same heating conditions. (b) Photograph of torch and sample. The distance between the torch tip and the sample is ~15 mm.

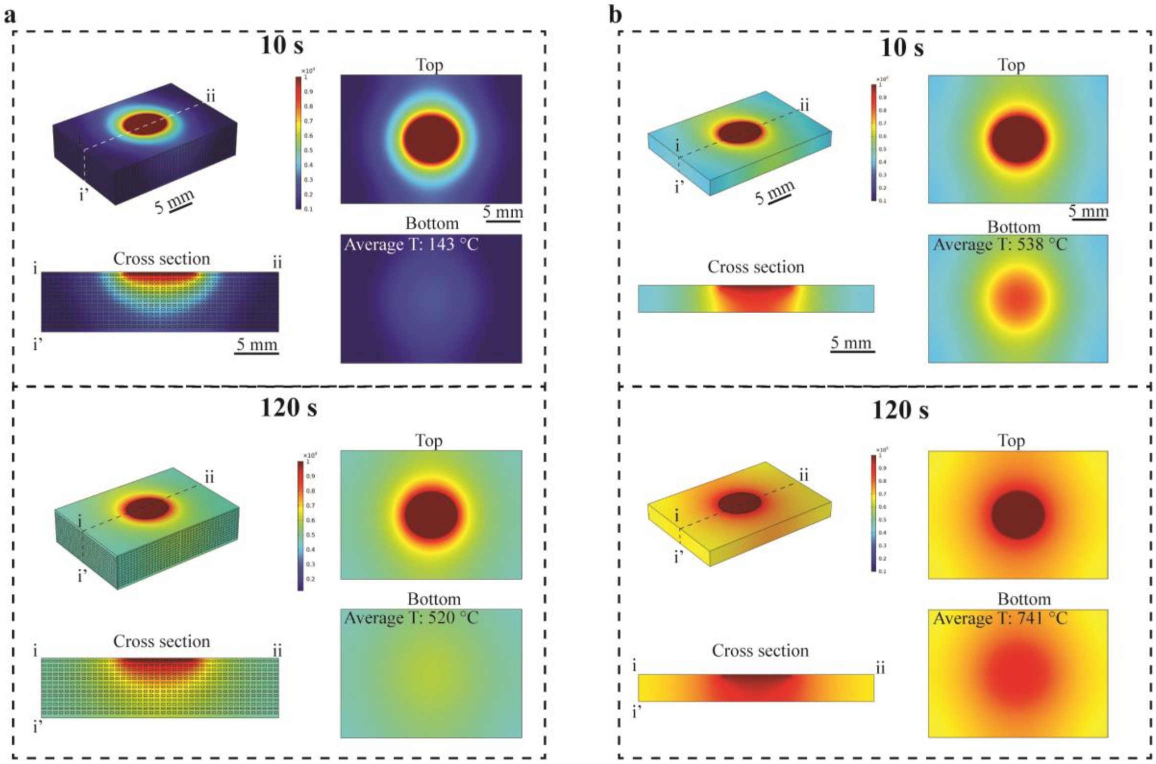

Supplementary Figure S3. Heat transfer modeling in the (a) lattice-core sandwich where cavity radiation is prevented via filling with a ceramic aerogel and (b) a dense plate with the same mass and area as the lattice-core sandwich for 10 s and 120 s. The modeling is implemented in the COMSOL Multiphysics package. The center circle on the top surface of the sample is set at 1000 ˚C at t=0 s, simulating a flame. Convective cooling (Convective Heat Transfer Coefficient h=20 W/m2·K [2]]) and surface-to-ambient radiation (emissivity ε=0.5[3]) are considered on the six outer surfaces of the sandwich. The convective cooling and cavity radiation on the internal surfaces are not considered given that the lattice-core sandwich is filled with aerogel (k=0.024-0.083 (W/m·K)) [4], which has a negligible heat transfer contribution in the current modeling. The result shows that the bottom surface of the lattice-core sandwich has a much lower average temperature (143 ˚C for 10 s and 519 ˚C for 120 s) than that of the bulk sample with the same mass (538 ˚C for 10 s and 741ºC for 120 s). Also, the peak temperature at the bottom surface is much reduced for the sandwich (286 ˚C for 10 s, 591 ˚C for 120 s) as compared to the dense plate (833 ˚C for 10 s, 907 ˚C for 120 s).

Supplementary Table S2. Oxygen, nitrogen, and carbon concentrations (as measured from IGF and combustion analysis) for samples after sintering at 1400 ˚C for 18 h, water quenching, and compressive deformation at 900 ˚C.

Sample	O (wt.%)	N (wt.%)	C (wt.%)
Sintered at 1400 ˚C/ 18 h	0.996	0.047	0.102
Water quenched	0.996	0.042	0.170
Compressed at 900 ˚C	0.904	0.047	0.102

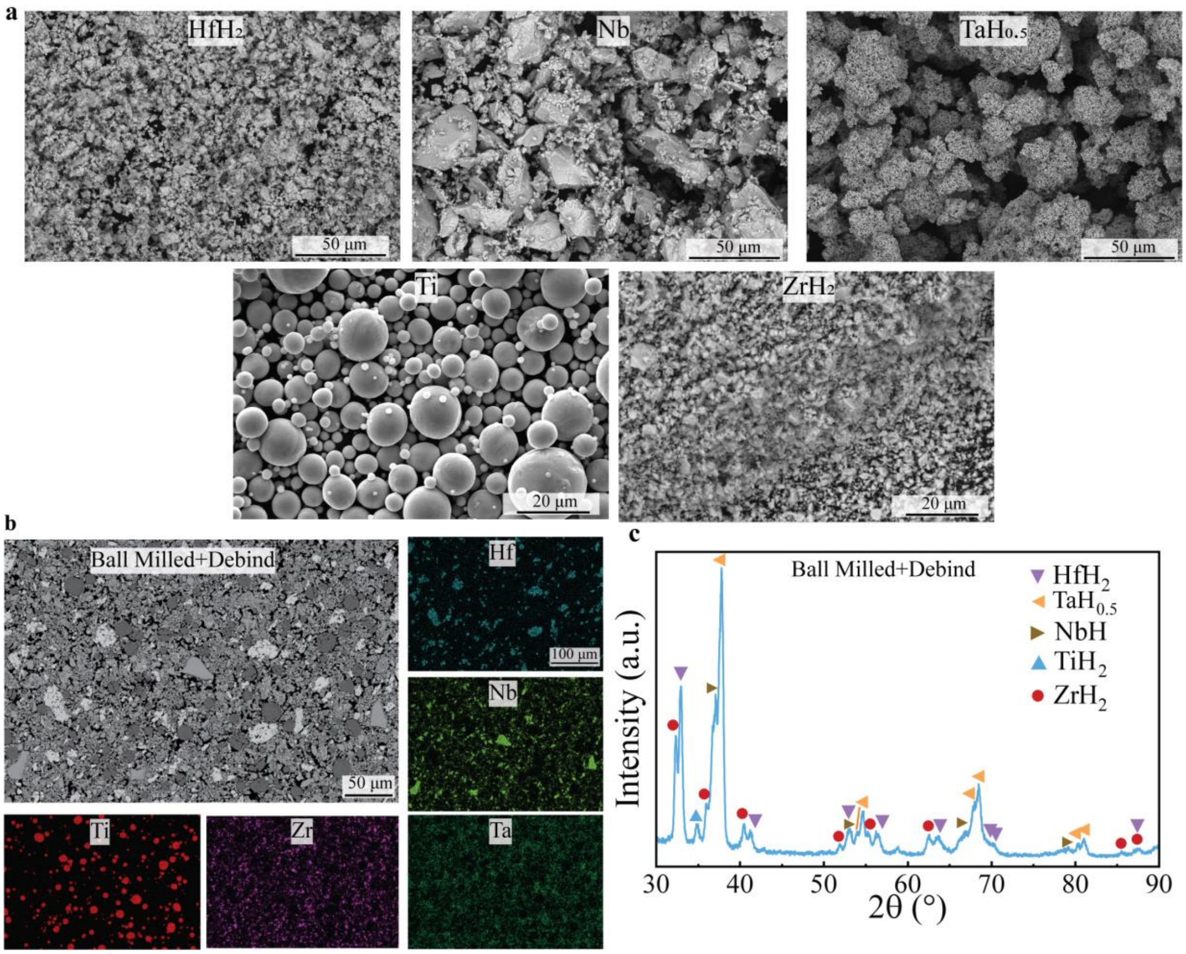

Supplementary Figure S4. (a) SEM-BSE micrographs of the HfH_2, Nb, $TaH_{0.5}$, Ti, ZrH_2 powders. (b) SEM-BSE and EDS micrographs of cross-section of ink ingot after ball milling and de-binding at 450 ˚C for 30 min under H_2. (c) corresponding XRD spectrum showing original hydride phases. From the XRD, the Nb and Ti powders are hydrogenated during the debinding process, performed under flowing H_2.

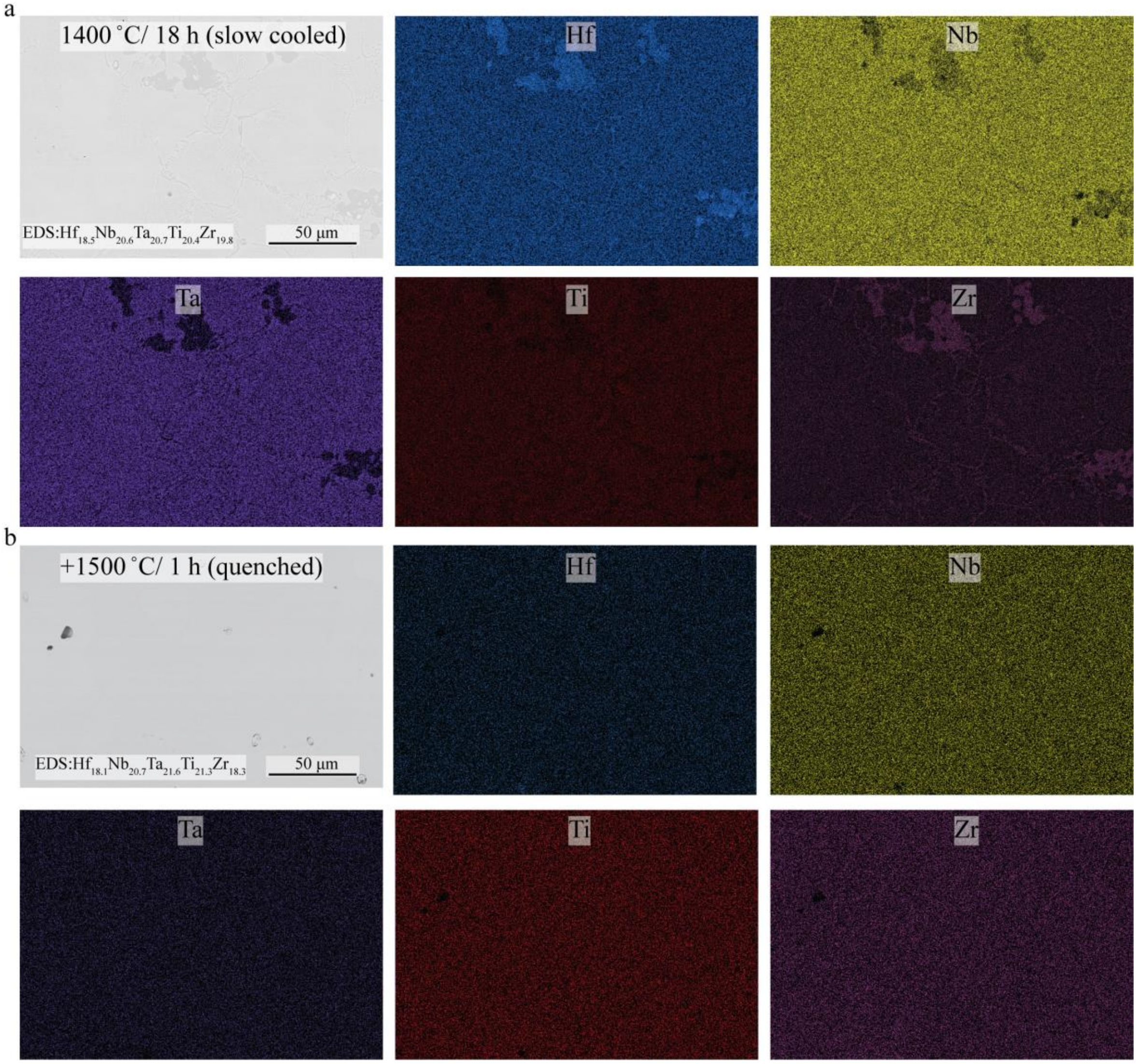

Supplementary Figure S5. SEM-BSE micrographs and corresponding EDS elemental maps for cross-sections of (a) ink ingot sample after sintering at 1400 ˚C/18 h and (b) after quenching from 1500 ˚C/1 h, showing that the distribution of five elements is near uniform in the quenched sample. The composition from EDS is labeled.

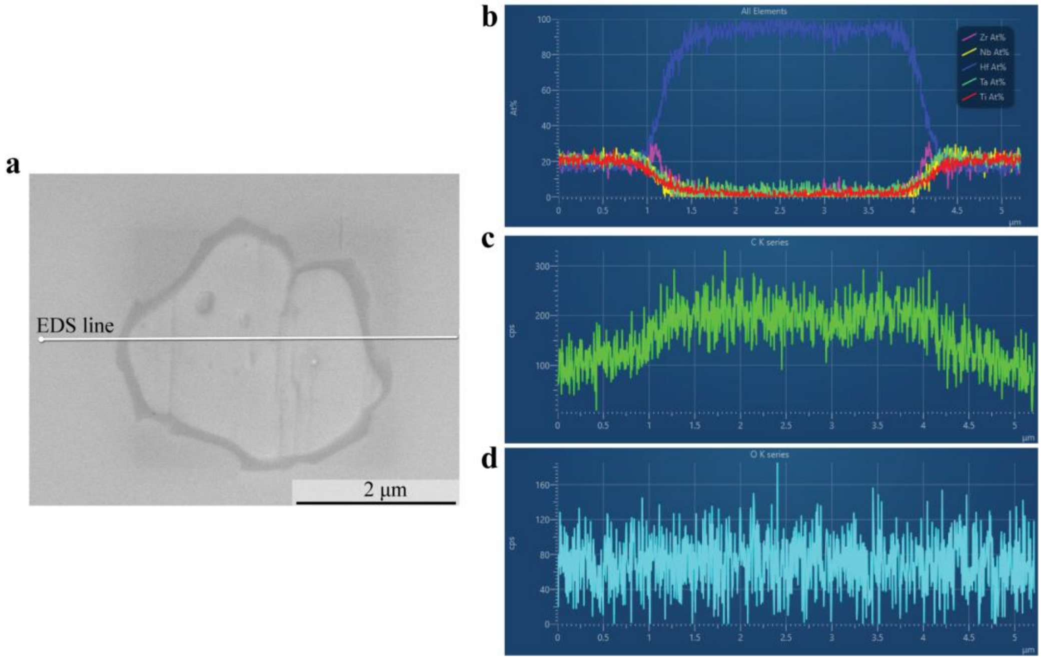

Supplementary Figure S6. SEM-BSE micrograph (a) and corresponding EDS line scan (b-d) for a HfC particle in the sample after quenching from 1500 ˚C/1 h. (b) The content of Hf, Nb, Ta, Ti, and Zr along the EDS line after excluding the O and C. (c) The EDS signal intensity of C along the EDS line. (d) The EDS signal intensity of O along the EDS line. The EDS line scan shows that this particle is enriched with Hf and C.

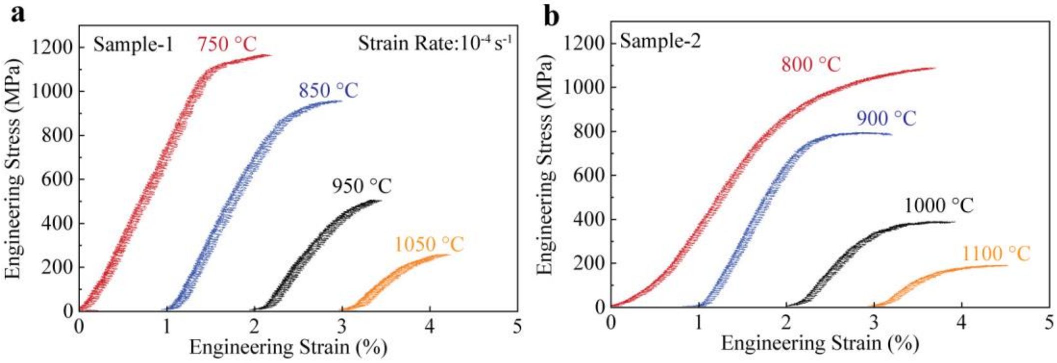

Supplementary Figure S7. Four load-unload compression tests of two sintered bulk samples (1400 ˚C/18 h) performed at (a) 750 - 1050 ˚C and (b) 800 - 1100 ˚C with a strain rate of 10^{-4} s^{-1} under flowing argon (99.999%). The curves are shifted by 1% on the x axis.

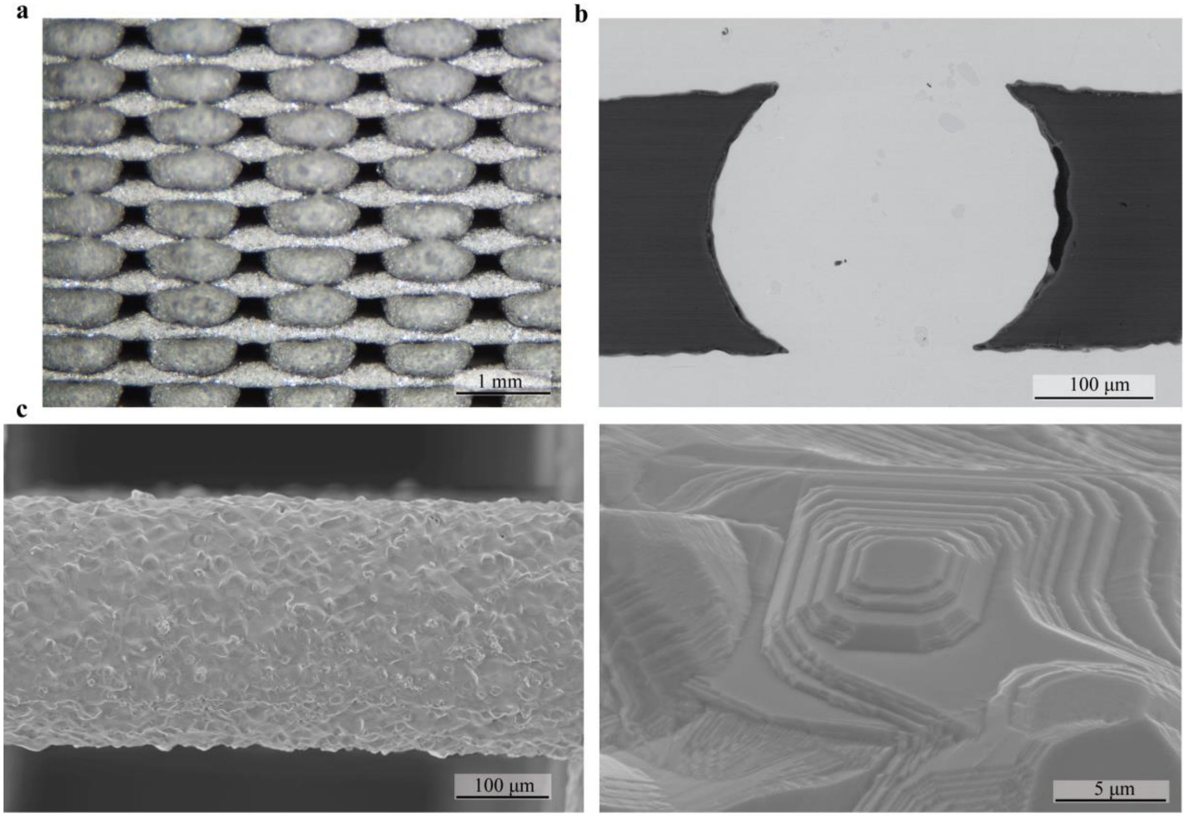

Supplementary Figure S8. (a) Photograph of the side face of a 3D-printed and sintered (1400 ˚C/18 h) lattice. No sagging is observed for the horizontal strut in the lattice. (b) SEM-BSE micrograph of cross-section for 3D printed and sintered lattice, which is consistent with microstructure shown in Figure 2 (c); pristine metallurgical bonding is observed between strut layers. (c) SEM-SE micrograph (left) and enlarged micrograph (right) of surfaces for a sintered filament. It exhibits a much smoother surface than struts in a lattice fabricated by LPBF from much coarser Ti-6Al-4V powders [5]. The terraced surface in the enlarged micrograph is consistent with surface diffusion or vapor phase transport, forming planes with low surface energy during sintering.

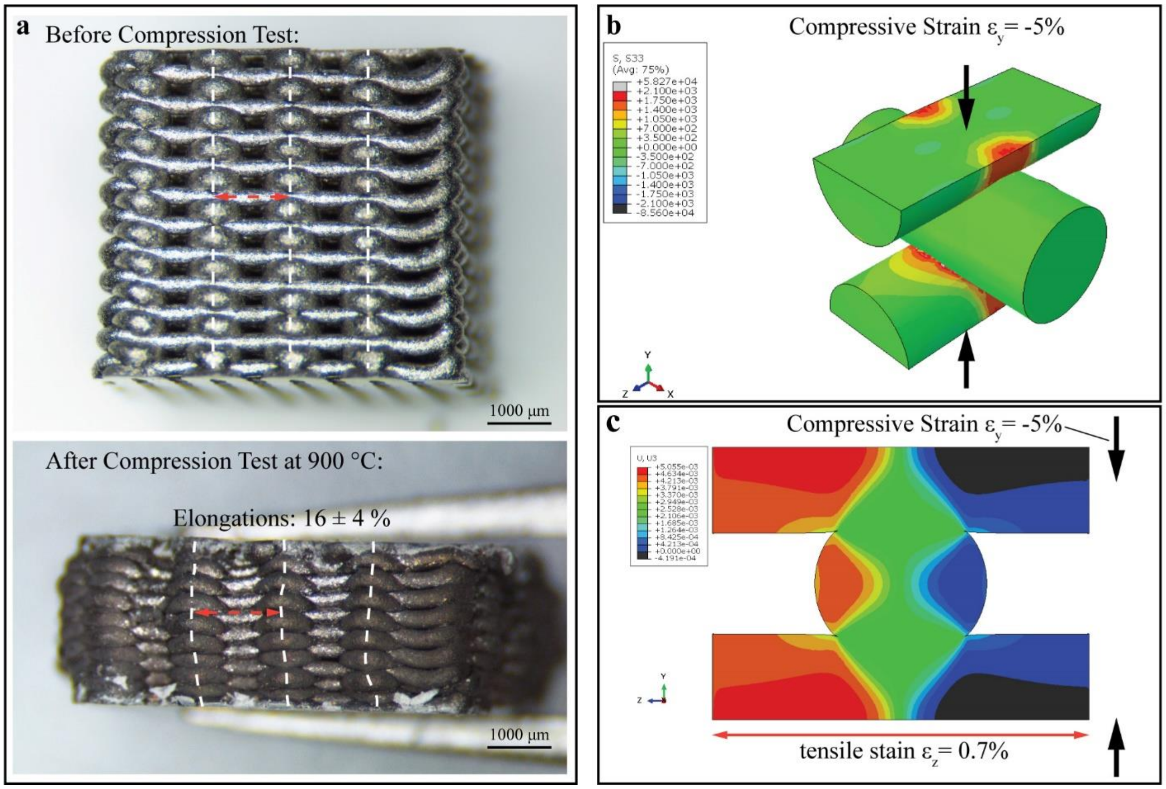

Supplementary Figure S9. (a) Macrographs of the side face of 3D-printed and sintered lattice before and after uniaxial compression deformation at 900 ˚C. After uniaxial compression of the lattice in the vertical direction, the horizontal struts are elongated by 16 ± 4% (as marked by white dotted lines), via the same volume-conservation mechanism observed in dense materials, where uniaxial compression leads to tensile strains in the cross-section of the material ("flattening"). Elongation of these struts without cracking or fracture indicates high tensile ductility at high temperatures. (b) Mechanical modeling for a representative volume element of a 0/90˚ cross-ply lattice structure, where stress along the z direction (σ_{zz} or S33) is mapped. The modeling is implemented in Abaqus CAE (2022). The details of boundary conditions can be found in Ref [6]. The elastic modulus is 90 GPa, and Poisson's ratio is 0.26 [7]. The tensile hardening behavior of HfNbTaTiZr at room temperature [8] is used here for illustrative purposes with a yield strength of 1682 MPa (estimated by 1/3 H_v [9]). (c) Displacement along the horizontal z direction (U3) after 5% compression strain in the vertical y direction. The struts show a tensile elongation along the z direction of about 0.7 %, which is consistent with the tensile stress state in the z direction shown in (b).

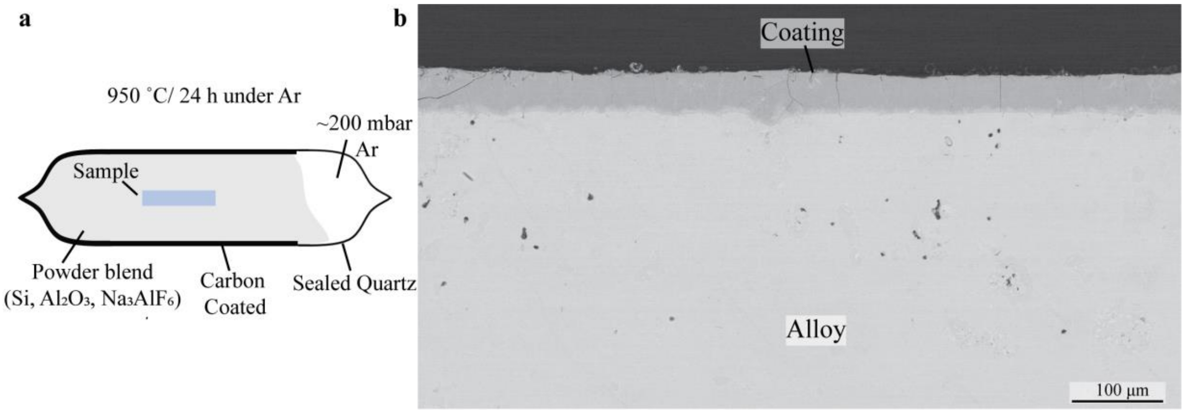

Supplementary Figure S10. (a) Schematic illustration of the pack used for silicide coating. (b) Low magnification SEM-BSE micrograph of cross-section of sintered and silicide-coated ink ingot sample; coating is uniform and ~45 um thick.

Supplementary References:

[1] C. Ho, R. Powell, P. Liley, *J. Phys. Chem. Ref. Data* **1974**, *3*, 11974.
[2] P. Kosky, R. Balmer, W. D. Keat, G. Wise, Exploring engineering: an introduction to engineering and design, Academic Press, 2012.
[3] A. El Bakali, R. Gilblas, T. Pottier, A. Lieurey, Y. Le Maoult, J. Alloys Compd. 2021, 889, 161545.
[4] Y. Chen, L. Zhang, C. He, R. He, B. Xu, Y. Li, Aerospace Science and Technology 2021, 111, 106539.
[5] E. Maleki, S. Bagherifard, M. Bandini, M. Guagliano, *Addit. Manuf.* **2021**, *37*, 101619.
[6] D. Zhang, C. Kenel, D. C. Dunand, Acta Mater. 2022, 238, 118187.
[7] G. Laplanche, P. Gadaud, L. Perrière, I. Guillot, J. P. Couzinié, J. Alloys Compd. 2019, 799, 538.
[8] L. H. Mills, M. G. Emigh, C. H. Frey, N. R. Philips, S. P. Murray, J. Shin, D. S. Gianola, T. M. Pollock, Acta Mater. 2023, 245, 118618.
[9] D. Tabor, The hardness of metals, Oxford university press, 2000.
